# Mitochondria-centric bioenergetic characteristics in cancer stem-like cells

**DOI:** 10.1007/s12272-019-01127-y

**Published:** 2019-02-15

**Authors:** Min-Kyue Shin, Jae-Ho Cheong

**Affiliations:** 1 Yonsei University College of Medicine, Seoul, Korea; 2 Department of Surgery, Yonsei University Health System, Yonsei University College of Medicine, 50 Yonsei-ro, Seodaemun-gu, Seoul, 120-752 Korea; 3 Yonsei Biomedical Research Institute, Yonsei University College of Medicine, Seoul, Korea; 4 Brain Korea 21 PLUS Project for Medical Science, Yonsei University College of Medicine, Seoul, Korea; 5 Department of Biochemistry & Molecular Biology, Yonsei University College of Medicine, Seoul, Korea; 6 Department of Biomedical Systems Informatics, Yonsei University College of Medicine, Seoul, Korea

**Keywords:** Bioenergetics, Cancer metabolism, Cancer evolution, Cancer stem cell, Mitochondria, Fatty acid oxidation, β-Oxidation

## Abstract

Metabolic and genotoxic stresses that arise during tumor progression and anti-cancer treatment, respectively, can impose a selective pressure to promote cancer evolution in the tumor microenvironment. This process ultimately selects for the most “fit” clones, which generally have a cancer stem cell like phenotype with features of drug resistance, epithelial-mesenchymal transition, invasiveness, and high metastatic potential. From a bioenergetics perspective, these cancer stem-like cells (CSCs) exhibit mitochondria-centric energy metabolism and are capable of opportunistically utilizing available nutrients such as fatty acids to generate ATP and other metabolic substances, providing a selective advantage for their survival in an impermissible environment and metabolic context. Thus, diverse therapeutic strategies are needed to efficiently tackle these CSCs and eliminate their advantage. Here, we review the metabolic and bioenergetic characteristics and vulnerabilities specific to CSCs, which can provide an unprecedented opportunity to curb CSC-driven cancer mortality rates. We particularly focus on the potential of a CSC bioenergetics-targeted strategy as a versatile therapeutic component of treatment modalities applicable to most cancer types. A cancer bioenergetics-targeted strategy can expand the inventory of combinatorial regimens in the current anti-cancer armamentarium.

## Introduction: unique aspects of cancer metabolism

Research on cancer cell-specific metabolism has exponentially increased in recent years, with recognition of the discovery of potential therapeutic targets for refractory cancers. This field has illuminated novel aspects of cancer cell biology related to the metabolic reprogramming that occurs during tumorigenesis and metastasis, which has even been referred to as a hallmark of cancer (Hanahan and Weinberg 2011). Subsequently, common features of cancer metabolism have been described, emphasizing the opportunistic utilization of nutrients available from the nutrient-poor tumor microenvironment (TME), with particular attention paid to deregulated nutrient uptake and various opportunistic modes of nutrient acquisition (Pavlova and Thompson 2016). The metabolic pathways that limit cancer progression and the context specificity of cancer metabolism have also been identified (Vander Heiden and DeBerardinis 2017). Several metabolic products, including ATP, NADPH, tricarboxylic acid (TCA) cycle intermediates, nucleotide bases, and electron acceptors, are considered to be limiting materials for cancer cell proliferation. Moreover, the TME, cell lineage, and interactions with benign cells determine the metabolic specificity of cancer cells. Given the crucial roles of metabolic reprogramming in cancer metastasis and therapy resistance, the knowledge accumulated from studies on cancer metabolism could be exploited to improve cancer therapy. Indeed, metabolic vulnerabilities are perceived as novel therapeutic targets of cancer, since metabolic reprogramming has been shown to direct cancer cells toward alternative and specific metabolic enzymes or pathways. For example, it was recently revealed that gastric cancer with epithelial-mesenchymal transition (EMT) gene expression can be targeted by the nicotinamide phosphoribosyltransferase (NAMPT) inhibitor FK866, since it relies on NAMPT for its nicotinamide adenine dinucleotide (NAD) metabolism due to the loss of nicotinic acid phosphoribosyltransferase (NAPRT), a compensatory enzyme for NAD biosynthesis salvage pathway (Lee et al. 2018). In addition, cancer metabolism enables discovery of novel diagnostic markers, which can be directly translated into clinical practice. For example, F-18 fluorodeoxyglucose (FDG) positron emission tomography is a representative diagnostic technology that detects cancer through metabolite-based imaging. Therefore, it is essential to explore the roles of each metabolic pathway in specific tumor progression stages or tumor subtypes for clinical translation (Martinez-Outschoorn et al. 2017).

To promote research in this field toward clinical application, in this review, we focus on the bioenergetics characteristics of cancer stem-like cells (CSCs) and highlight the therapeutic opportunities that can arise by identifying metabolic vulnerabilities specific to this deadly subpopulation. In particular, we provide an overview of recent findings implicating mitochondria-centered bioenergetics during cancer malignant evolution in selective tumor environmental context. We then describe the specific metabolic pathways and key molecules enabling metabolic stress adaptation during cancer progression. Finally, we introduce new strategies to pharmacologically intervene with cellular bioenergetics in malignant cancers.

## Cancer progression and metabolic adaptation

Cancer progresses in the defined and adaptive microenvironement. Both the interaction between heterogeneous subpopulations within a given cancer type and that between the cancer cells and surrounding microenvironment influence tumor progression, metastasis, and ultimate drug resistance (Tabassum and Polyak 2015). Genetic heterogeneity between individual tumors and intratumoral cells (Gillies et al. 2012) shapes such an interactive and adaptive landscape of cancer which imposes a major limitation for advances in effective cancer treatment. Moreover, there is inherent functional heterogeneity within a single genetic clone, further contributing to ultimate therapeutic failure. Together, regardless of apparent gene-level differences, tumor cells display substantial variation in growth dynamics and in the response to therapy. For example, a slowly proliferating population has been shown to retain tumor propagation potential after chemotherapy (Kreso et al. 2013). Consequently, it is crucial to explore the functional heterogeneity of cancer cells at the level beyond genetic mutations. In this regard, the reprograming of cancer metabolism caused by reversible activation of metabolic pathways is now attracting increased research attention (Hensley et al. 2016).

From a metabolic perspective, bioenergetic fuel production is mediated by the metabolic reprogramming of both cancer and non-cancer cells in the tumor microenvironment (TME) (Wolpaw and Dang 2018). Not only metabolic cross-talk between tumor and stromal cells, metabolic reprogramming mediated by bacterial oncoproteins has been shown to directly contribute to carcinogenesis. *Helicobacter pylori* cytotoxin-associated gene A (CagA) increases reactive oxygen species (ROS) production and activates the transcription factor hypoxia inducible factor 1α (HIF-1α), which in turn facilitates the metabolic changes that help cancer cells survive under hypoxia and glucose deprivation (Lee et al. 2017). Thus, it is of great importance to understand metabolic reprogramming in cancer cells as a means of adaptive process in the context of the selective tumor microenvironment.

## CSCs arise in the face of metabolic stress

Cancer cells develop their malignant characteristics when undergoing metabolic adaptations in the face of metabolic stress. As the cancer progresses, the TME becomes increasingly hypoxic and nutrient-deprived, accompanied by a reduction in pH, and these conditions show both spatial and temporal heterogeneity. Under hypoxia or glucose deprivation, activation of the energy sensor 5′-AMP-activated protein kinase (AMPK) inhibits anabolic processes (Zadra et al. 2015). Moreover, metabolic stress promotes the emergence of CSCs, which are the most evolved distinct subpopulations in a tumor. CSCs are characterized by stem-like malignant behaviors, and are the causes of relapse, metastasis, and drug resistance of a cancer. EMT, which enables the acquisition of cancer stemness, is associated with catabolic reprogramming during metabolic stress (Cha et al. 2015). Long-term nutrient deprivation of the TME facilitates the Wnt-dependent transition of non-stem cancer cells toward a stem-like cell state (Lee et al. 2015a). Furthermore, Wnt signaling is associated with reprogramming of NAD metabolism (Lee et al. 2016b). CSCs express various protein markers such as CD44, Aldehyde dehydrogenases (ALDHs, e.g. ALDH1A1) and CD133, and these markers serve to isolate CSCs from the bulk tumor cell population. Importantly, ALDHs are regulated by β-catenin/TCF, effector molecules of Wnt pathway (Cojoc et al. 2015), and are responsible for resistant to anti-cancer treatment (Raha et al. 2014). Among diverse metabolic functions of ALDHs, ALDHs catalyze the conversion of aldehyde to carboxylic acid and the production of NADH which contributes to ATP production (Kang et al. 2016). In addition, CSCs express sarco/endoplasmic reticulum Ca^2+^-ATPase to avoid Ca^2+^-dependent apoptosis under glucose deprivation (Park et al. 2018b). Together, this metabolic reprogramming and altered dependency on specific pathways provide a selective advantage for the survival of CSCs. Therefore, targeting these metabolic adaptations of CSCs should provide new opportunities to overcome malignant tumors.

## Mitochondria-centered cancer bioenergetics

Mitochondrial bioenergetics plays a central role in cancer metabolism, thereby serving as the driving force for cancer progression. Cells make use of different nutrient molecules such as glucose, glutamine, and fatty acids (FAs) according to their specific anabolic and catabolic needs depending on the cell state, i.e., quiescence, pluripotency, and proliferation (Stanley et al. 2014). This selective nutrient utilization results in bioenergetic reprogramming to maintain the differentiation and proliferation of cells under metabolic stress. Aerobic glycolysis, or the Warburg effect, may be the most well-known feature of cancer bioenergetics. However, many types of cancer cells rely on mitochondrial respiration, displaying remarkable versatility in their bioenergetic profiles (Alam et al. 2016). Furthermore, the mitochondria in cancer cells play unique and important roles beyond their key bioenergetics function, such as biosynthesis, redox homeostasis, retrograde signaling with the nucleus, regulation of the microenvironment, and modulation of the immune system (Vyas et al. 2016). Notably, the importance of mitochondrial function in CSCs and its contribution to malignant phenotypes—metastasis and treatment resistance—are gradually becoming disclosed (Seo et al. 2014; Jeon et al. 2016; Sancho et al. 2016). Meanwhile, mitochondrial biology and genetics are starting to be recognized as an important part of the Precancer Atlas, a precision medicine-based prevention effort integrating the fields of multi-omics and immunity, since disruption of mitochondrial respiration has potential as a cancer prevention strategy and changes in mtDNA largely influence cancer risk (Spira et al. 2017). Thus, understanding the key factors that regulate mitochondrial function and bioenergetic flexibility in cancer might help to identify novel therapeutic targets (Obre and Rossignol 2015).

**Fig. 1:**
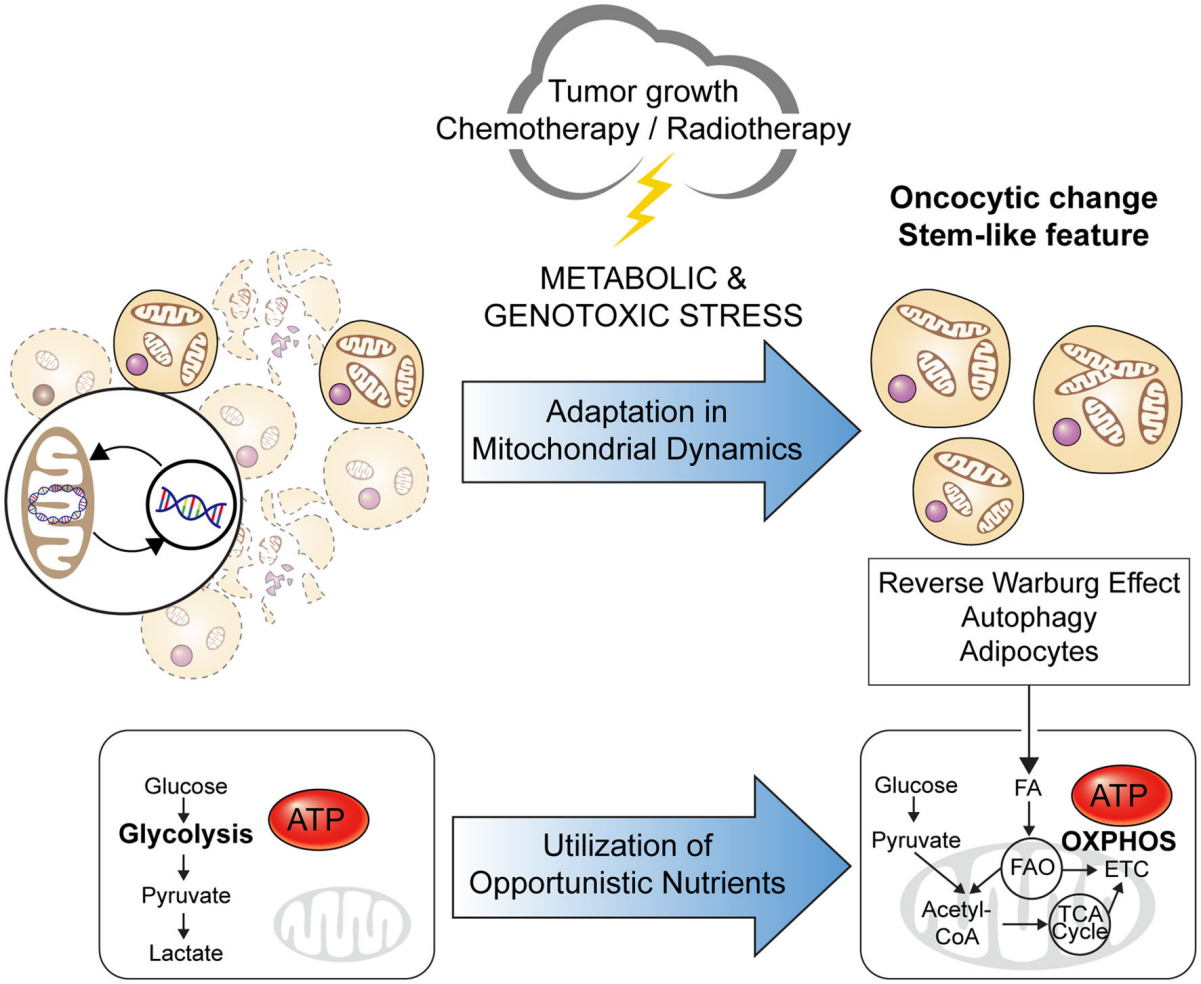
Changes in mitochondrial dynamics during cancer evolution. Metabolic stressors such as nutrient deprivation due to tumor growth, as well as chemotherapy and radiotherapy act as a selection pressure to cancer cells. Metabolic adaptation through increased mitochondrial biogenesis and fusion—which result in numerous enlarged, elongated, and interconnected mitochondria—augments the oxidative capacity and ATP production in cancer cells, thereby enabling their survival. This process has been supported by the discovery of an oncocytic change and cancer stem cells (CSCs), both of which are associated with the malignant phenotypes of a cancer

Mitochondrial dynamics is one of the main factors contributing to regulating mitochondrial bioenergetics. The mitochondrial architecture, including the shape, size, and localization, regulates energy and metabolic homeostasis, and its deregulation is implicated in cancer metabolism. Under intracellular stress and a condition of nutrient limitation, alteration of the mitochondrial architecture and dynamics enable the metabolic adaptation and evasion of cell death programs in cancer cells to ultimately support cancer cell proliferation, migration, and drug resistance (Senft and Ronai 2016; Trotta and Chipuk 2017). Indeed, an increased mitochondrial mass and elongated morphology are distinctive features of CSCs, allowing for their anchorage-independent growth and chemo-resistance. Specifically, mitochondrial biogenesis, fusion, and cristae modulation regulate mitochondrial respiration, enabling CSCs to adapt to energy stress (De Luca et al. 2015; Li et al. 2017). For example, the phenomenon of “oncocytic change” was discovered among residual cancer cells after neoadjuvant chemoradiotherapy, and was associated with a poor prognosis (Ambrosini-Spaltro et al. 2006; Hong et al. 2016). An oncocytic change is defined as cells with an enlarged, eosinophilic (oxyphilic), and finely granular morphology that contain abundant mitochondria, and has been proposed to occur as a compensatory response to reduced mitochondrial activity under oxidative stress (Guaraldi et al. 2011), representing a typical example of deregulated mitochondrial dynamics in cancer cells and the consequent influence on cancer progression and treatment responses (Fig. 1).

Another key factor regulating mitochondrial bioenergetics is mitochondrial DNA (mtDNA) itself, which contains genes of the electron transport chain (ETC). Various mtDNA abnormalities and the associated mitochondrial dysfunction have been demonstrated to support cancer progression, contributing to tumorigenesis, metastasis, and treatment resistance (van Gisbergen et al. 2015). Moreover, horizontal transfer of mtDNA from the TME to tumor cells with compromised mitochondrial function can initiate tumorigenesis by restoring respiration. When an mtDNA-deficient tumor cell line was injected subcutaneously into mice, the primary tumor cells showed delayed tumor growth, but tumor formation was possible after the cells acquired mtDNA from the host cells. Similar recovery of respiratory function has been demonstrated in circulating tumor cells and metastatic tumor cells (Tan et al. 2015). Moreover, cross-talk between mitochondrial and nuclear genomes contribute to AMPK-dependent metabolic adaptation under glucose deprivation (Kim et al. 2018). Collectively, these findings indicate the importance of mitochondrial function for tumor initiation and progression.

## Oxidative phosphorylation (OXPHOS) confers malignant phenotypes

The two major pathways of energy metabolism in eukaryotes are glycolysis and mitochondrial OXPHOS; the latter process, which is mediated by ETC, involves supply of reducing equivalents from TCA cycle or fatty acid oxidation (FAO; also known as β-oxidation) (Erecinska and Wilson 1982). However, only the glycolysis pathway has been a focus of cancer research, because the Warburg effect has long been considered as the doctrine of cancer metabolism. Emerging evidence demonstrating that cancer cells make full use of other bioenergetic pathways has resulted in more research focus on these other pathways in recent years. For example, it has been demonstrated that cancer cells can acquire metabolic plasticity by gaining a hybrid glycolysis/OXPHOS phenotype. That is, cancer cells can switch between these two bioenergetic pathways to improve their survival under harsh conditions. The potential role of metabolic heterogeneity and plasticity in cancer metastasis and therapy resistance is also recently becoming recognized (Jia et al. 2018), and studies on CSCs have highlighted the particular significance of OXPHOS and FAO in many malignant tumor types.

Cancer cells become dependent on OXPHOS during their progression, while concurrently acquiring treatment resistance. Slow–cycling stem-like cancer cells, selected by cytotoxic agents, are drug-resistant and have been suggested to have accelerated highly energy-consuming metabolic pathways. In contrast to the classic notion of rapidly dividing cancer cells that primarily depend on anaerobic glycolysis, this subpopulation of quiescent cells depends on mitochondrial respiration to meet their high energy demands. Therefore, OXPHOS can be a druggable target to expose the vulnerability of drug-resistant cancers, which should be synthetically lethal along with cytotoxic therapies (Wolf 2014). Chemotherapy was shown to induce OXPHOS in several cancer models, thereby conferring them with drug resistance. Moreover, this type of bioenergetic adaptation enables cancer cells to survive under glucose deprivation (Dar et al. 2017). Likewise, targeted treatment with BRAF and PI3K inhibition resulted in OXPHOS addiction in melanoma and glioma cells, ultimately conferring them with drug resistance (Haq et al. 2013; Caino et al. 2015). In particular, PI3K therapy induced the subcellular localization of mitochondria with consequent spatiotemporal OXPHOS enhancement that fueled tumor cell invasion. The activity of ATP-binding cassette (ABC) transporter, which sustains chemoresistance by exporting cytotoxic agents, has been associated with OXPHOS activity (Porporato et al. 2018). In addition, radiation treatment could increase the OXPHOS efficiency by decreasing mitochondrial proton leakage in breast CSCs, which displayed reliance on OXPHOS. Furthermore, radio-resistance was correlated with the mitochondrial reserve capacity in glioma stem cells, which were also deemed to be OXPHOS-dependent (Vlashi et al. 2014).

Moreover, enhanced OXPHOS contributes to the stemness and metastatic potential of cancer cells. OXPHOS is verified as the main bioenergetic pathway in several types of CSCs, supporting their differentiation and survival under metabolic stress (Jang et al. 2015). ATP production fuels membrane dynamics, while mitochondrial superoxide production promotes tumor cell migration. In addition, subcellular mitochondrial trafficking has emerged as a central regulator of tumor cell motility, invasion, and metastasis, playing a crucial role in the metabolic adaptation to microenvironmental stress (Altieri 2017). Drug-resistant lung cancer cells exhibit increased mitochondrial membrane potential and show upregulated expression of ETC genes, which was associated with enhanced migration and invasion that was impeded by the inhibition of mitochondrial activity (Jeon et al. 2016). Moreover, OXPHOS mediates the metabolic symbiosis between cancer cells and cancer-associated stromal cells within the TME. The newly proposed theory of the “Reverse Warburg Effect” describes a two-compartment model in which glycolytic stromal cells provide fuel for OXPHOS in cancer cells. This metabolic coupling has been implicated in the proliferation and growth of cancer cells, as well as in the recurrence, metastasis, and drug resistance of cancer (Wilde et al. 2017). These facts indicate that shutting off OXPHOS would be a potential strategy to overcome the malignant phenotypes of cancers.

Along with the increasing recognition of OXPHOS as the major bioenergetic pathway in CSCs, its regulators are emerging as novel therapeutic targets. Peroxisome proliferator-activated receptor gamma coactivator 1-alpha (PGC-1α) is a transcriptional co-activator that acts as a master regulator of mitochondrial metabolism. In cancer cells, PGC-1α serves as a stress sensor that is activated under nutrient limitation, oxidative stress, and chemotherapy to facilitate mitochondrial biogenesis, OXPHOS, FAO, and ROS detoxification. PGC-1α has been reported to have both oncogenic and tumor-suppressive features: its expression is downregulated in the early stage of carcinogenesis, thereby inducing glycolysis to play an anticancer role, whereas it is upregulated in the late stage of cancer progression, contributing to the metabolic plasticity that facilitates tumor cell growth, metastasis, and drug resistance (Mastropasqua et al. 2018). In addition, insulin-like growth factor 2 mRNA-binding protein 2 (IMP2) is an RNA-binding protein that mediates transcript processing in cells, and it regulates the processing of key subunits in mitochondrial complexes along with their assembly to ultimately promote the survival, proliferation, and migration of cancer cells. Importantly, IMP2 maintains OXPHOS in CSCs to enable their self-renewal (Cao et al. 2018). A recent transcriptome analysis comparing different cancer molecular subtypes revealed that a mesenchymal subtype of gastric cancer with a poor prognosis was associated with increased activation of the insulin-like growth factor 1 pathway, further suggesting the bioenergetics significance of cancer behavior (Oh et al. 2018). Similarly, an Epstein-Barr virus-associated subtype of gastric cancer with a good prognosis was associated with reduced metabolic activity and energy production (Sohn et al. 2017). In addition, forkhead box P3, a transcription factor of regulatory T cells (Tregs), was shown to suppress glycolysis and enhance OXPHOS in low-glucose, lactate-rich environments, promoting the metabolic adaptation of Tregs and ultimate immune tolerance in the TME (Angelin et al. 2017). Thus, these regulators of OXPHOS constitute key mechanisms of metabolic reprogramming in malignant cancers, and would thus serve as legitimate targets to hinder cancer progression.

## FAO as a metabolic stress adaptation

Along with OXPHOS, the crucial role of FAO in cancer is only recently coming to light. Importantly, increased FAO activity was shown to promote oncogenesis. Remodeling of FA metabolism and FAO induction were proven to be involved in pancreatic tumorigenesis driven by mutant G protein alphas. Acetyl coenzyme A (CoA) derived from FAO supports the transformation of intraductal papillary mucinous neoplasms to pancreatic ductal adenocarcinomas (PDACs). In addition, coordinated triglyceride synthesis and FAO have been described in KRAS-mutant lung cancer (Patra et al. 2018). Furthermore, some tumors have been shown to be highly dependent on FAO for their survival and proliferation. Overexpression of carnitine palmitoyltransferase 1 (CPT1), which catalyzes the rate-limiting step of FAO, has been correlated with cancer progression in numerous types of cancer. Notably, FAO has great capacity to fuel cancer cells under metabolic stress, since it is the major opportunistic source of ATP and NADPH (Qu et al. 2016). ATP production from FAO prevents anoikis in cancer cells undergoing loss of attachment from solid tumors to the extracellular matrix, which restricts glucose uptake and catabolism. For example, FAO was reported to rescue mammary epithelial cells from the ATP deficiency caused by matrix detachment when acquiring anchorage independence. Furthermore, the NADPH produced from FAO counteracts oxidative stress, facilitating cancer cell survival (Carracedo et al. 2013). Likewise, FAO can maintain the bioenergetics of Akt-expressing glioblastoma cells under glucose deprivation: stimulation of FAO protected the cells from glucose withdrawal-induced death, while inhibition of FAO hindered this effect (Buzzai et al. 2005). CSCs of epithelial ovarian cancer exploit OXPHOS and FAO to overcome glucose deprivation, and this metabolic trait was associated with the resistance to chemotherapy (Dar et al. 2017). The substitution of FAO for glucose catabolism has also been demonstrated in acidic pH-adapted cancer cells. Tumor acidosis decreased glycolysis and acetyl-CoA production from glucose oxidation so that FAO became the main source of acetyl-CoA, fueling the TCA cycle and enabling tumor cell proliferation (Corbet et al. 2016). This finding was supported by not only an increase in FA uptake but also the compartmentalization of FAO in the mitochondria and FA synthesis in the cytosol. Such concomitant occurrence of opposite metabolic pathways emphasizes the importance of overall FA metabolism in metabolic reprogramming. In addition, phospholipase D1 (PLD1)-regulated autophagy was shown to mediate cancer cell survival under glucose deprivation through FAO induction. PLD1 hydrolyzes membrane phospholipids, thereby supplying FAs for oxidation. Thus, inhibition of PLD1 suppressed FAO during glucose deprivation, depleting ATP while increasing ROS, which resulted in cancer cell death (Cai et al. 2016). In summary, cancer cells use FAO as an adaptive bioenergetic pathway to maintain their viability under metabolic stress, including nutrient deprivation and the harsh TME.

Many genes involved in FA metabolism are correlated with cancer metastasis, drug resistance, and relapse. This may reflect the dependency of CSCs on FAO for survival, which supports the reduction of lipotoxicity, efficient production of ATP in slow-cycling cells, and generation of acetyl-CoA for protein acetylation and FA synthesis (Kuo and Ann 2018). Long-chain fatty acyl-CoA synthetases, which facilitate the initial step of FA metabolism, are frequently deregulated in cancer, and their overexpression has been associated with a poor prognosis in cancer patients (Tang et al. 2018). High-grade clear cell renal cell carcinoma (ccRCC) exhibited dependence on FAO, while low-grade ccRCC exhibited dependence on glycolysis. Inhibition of FAO with etomoxir significantly decreased ATP production and exerted a cytotoxic effect only in high-grade ccRCC cells, whereas inhibition of glycolysis with 2-deoxy-d-glucose (2DG) impaired cell viability only in low-grade ccRCC cells (Bianchi et al. 2017). MYC-overexpressing triple-negative breast cancer (TNBC), the most aggressive subtype of breast cancer, also displayed reliance on FAO (Camarda et al. 2016). Furthermore, residual breast cancer cells after neoadjuvant treatment showed an increase in FA metabolism (Havas et al. 2017). Similar alterations in bioenergetics have been demonstrated in pancreatic cancer relapse. Both inhibition of OXPHOS and FAO abrogated survival and the spherogenic potential of dormant PDAC cells that survived *KRAS* oncogene ablation (Viale et al. 2014). These results indicate that inhibition of FAO has potential to suppress malignant cancer cells.

Adipocytes are important components of the TME that promote cancer growth via FAO and adipokine signaling. In addition, chronic inflammation in the adipose tissue exerts genotoxic stress that promotes tumorigenesis. Several types of cancer cells obtain energy from the adjacent adipose tissue, including breast, colon, and ovarian cancer cells, and leukemic CSCs (Lengyel et al. 2018). Such metabolic symbiosis enables cancer cell survival under nutrient deprivation and is associated with expression of CSC-related genes, while contributing to the aggressiveness of cancers, supporting metastasis and drug resistance. In particular, the direct role of adipocytes in cancer progression has been suggested from analysis of breast tissue, in which adipocytes accounted for the largest proportion of cells (Choi et al. 2018). FAs were shown to be released from lipolysis in adipocytes, and then transferred to the breast cancer cells, supporting their growth by fueling FAO. Moreover, exosomes secreted by adipocytes can promote the aggressive behavior of cancer cells through FAO induction (Lazar et al. 2016). Exosomes are cell-derived nanovesicles that allow for cell–cell communication and are implicated in cancer progression. Exosomes transfer proteins involved in FAO from adipocytes to melanoma cells, inducing FAO-dependent cell migration and invasion. Furthermore, the number and effect of adipocyte exosomes are amplified in obesity. Thus, targeting the metabolic symbiosis between cancer cells and adipocytes in the TME holds therapeutic potential.

## Molecular regulation of FAO in cancer cells

Identification of the regulators of FAO has further provided novel therapeutic targets, including enzymes, transcription factors, and genes. CPT1 is the enzyme that most directly catalyzes FAO, and it is also the most crucial and targetable enzyme in this pathway (Qu et al. 2016). CPT1 is a mitochondrial enzyme responsible for the delivery of long-chain FA from the cytosol to mitochondria, which is the rate-limiting step of FAO. The three isoforms of CPT1, CPT1A, CPT1B, and CPT1C, show a tissue-specific distribution, although their inhibition or depletion has similar anticancer efficacy, including suppression of proliferation, drug resistance, and neovascularization. CPT1 not only plays a crucial role in the production of ATP and NADPH but also regulates cancer cell apoptosis owing to its antagonistic interaction with Bcl-2 family members and clearance of cytotoxic lipids. AMPK frequently induces CPT1C in aggressive cancers to promote cancer cell survival under metabolic stress. Surprisingly, the tumor suppressor p53 and LKB1 were shown to mediate this metabolic plasticity in an AMPK-dependent manner. Consistently, the carnitine system is considered as the pivotal mediator of metabolic plasticity in cancer cells, with energetic and biosynthetic functions supporting cell viability and uncontrolled proliferation. Moreover, its effects on enzymatic and epigenetic levels have been described in association with the aggressiveness and metastatic potential of cancer (Melone et al. 2018). Collectively, these findings suggest CPT1 as one of the most promising druggable targets for cancer prevention and treatment.

AMPK is another important enzyme that regulates FAO, given its function as a major regulator of cellular bioenergetics (Jeon 2016). Specifically, AMPK triggers the metabolic adaptation of cancer cells under metabolic stress, while maintaining the production levels of ATP and NADPH. AMPK inhibits acetyl-CoA carboxylase, which generates malonyl-CoA. Since malonyl-CoA is an allosteric inhibitor of CPT1, activation of AMPK eventually increases CPT1 activity. Furthermore, AMPK activates PGC-1α and contributes to mitochondrial biogenesis and the maintenance of mitochondrial integrity. Therefore, inhibition of AMPK could be an effective strategy for treating advanced cancers with metabolic adaptation. By contrast, inactivation of AMPK could also facilitate carcinogenesis by activating mTORC1 and promoting genetic mutations, which highlights the context-dependent role of AMPK in cancer, including the stage of cancer progression. In addition, a recent study showed that the combination of suppressed AMPK activity and FAO sensitized cancer cells to a state of glucose deprivation (Pan et al. 2017). Therefore, the roles of AMPK in specific contexts should be confirmed, and methods to modulate its activity to cure cancer require further development.

The peroxisome proliferator-activated receptor (PPAR) family of nuclear receptors function as transcription factors, and they have recently been shown to play a role in cancer metabolism through FAO. Constitutive activation of PPARα activated FAO, and the subsequent production of ATP and ROS contributed to the pathogenesis of hepatocellular carcinoma (HCC) (Misra and Reddy 2014). PPARα regulates genes involved in mitochondrial functions, including FA transport and FAO, and its regulatory effect on CPT1 has been shown to promote oncogenic activity in several tumor tissues (Antonosante et al. 2018). Moreover, PPARα-mediated FAO has been associated with the aggressiveness of chronic lymphocytic leukemia (CLL), conferring immunosuppression and resistance to metabolic and cytotoxic stress (Tung et al. 2013). This in turn promoted resistance of CLL cells to glucocorticoid treatment, which targets cellular bioenergetics. PPARα inhibition had a cytotoxic effect on CLL cells and improved the therapeutic efficacy of glucocorticoid therapy, whereas activation of PPARα with its agonist fenofibrate was shown to suppress tumorigenesis in several studies, possibly due to the triggering of ineffective tumor metabolism (Lian et al. 2018). The switch of bioenergetics pathways from glycolysis to FAO has been suggested to decrease ATP production and increase ROS production. Furthermore, PPARγ has demonstrated both antiproliferative effects and tumorigenic potential depending on the tumor tissue and TME, which may be attributed to different effects at different stages of cancer progression and in different metabolic contexts. Therefore, the role of PPAR-activated FAO should also be further investigated with consideration of specific molecular partners and environments.

PPARs are co-regulated by PGC-1α to promote metabolic adaptation in specific cell types. Surprisingly, this mechanism was revealed to involve promyelocytic leukemia (PML), generally known as a tumor suppressor, which deacetylates PGC-1α, leading to activation of PPAR signaling to promote FAO, thereby supporting the survival, growth, and malignant phenotype of breast cancer cells (Tan et al. 2016). This PML-PPAR-FAO pathway was also shown to regulate the maintenance and asymmetric division of hematopoietic stem cells, and has been linked to the maintenance of stemness in CSCs (Ito et al. 2012).

**Fig. 2:**
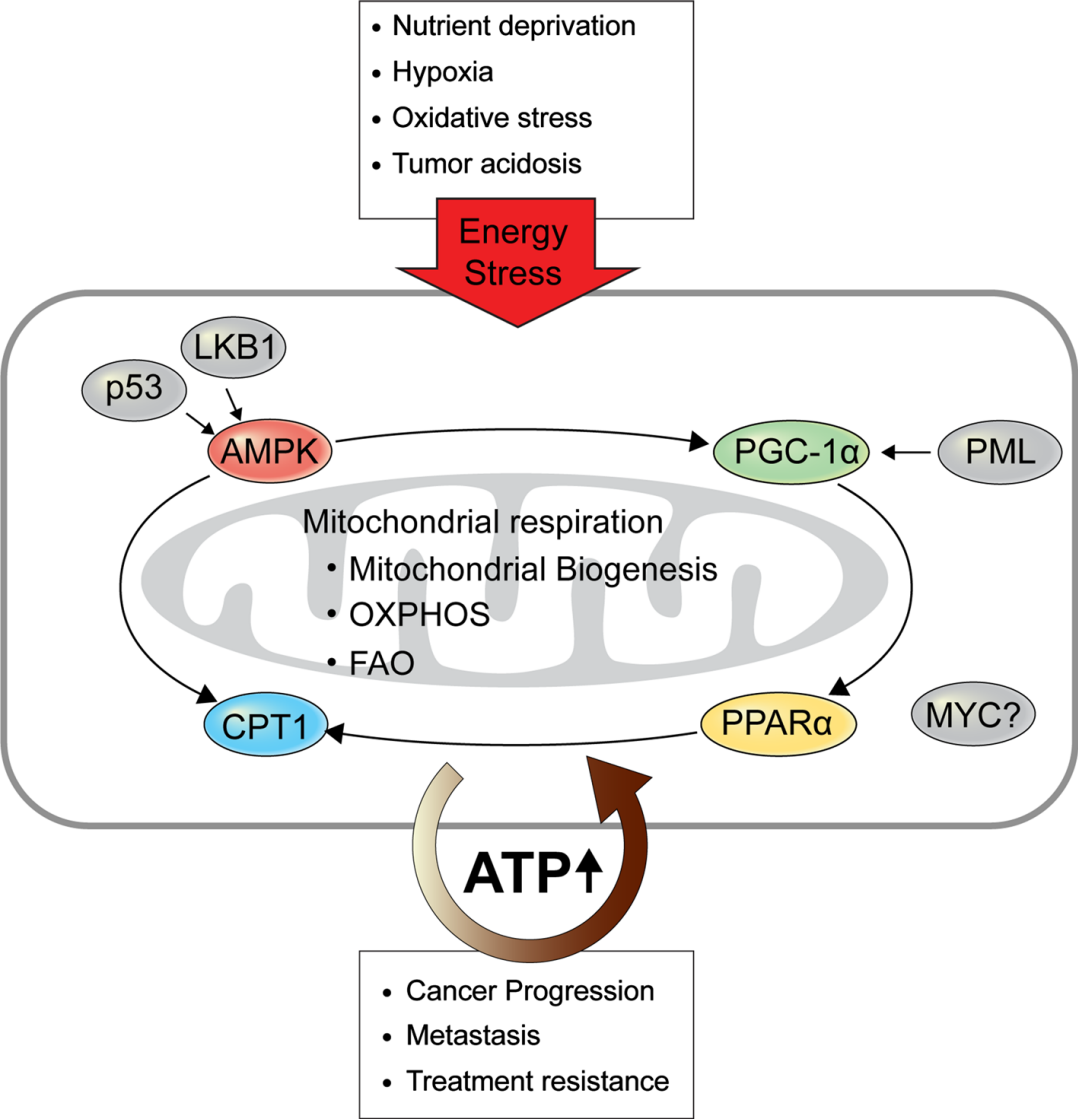
Regulators of OXPHOS and FAO. In response to energy stress, cancer cells activate PGC-1α and AMPK signaling, which are mediated by the tumor suppressor genes PML, p53, and LKB1. Both signaling pathways in turn augment mitochondrial biogenesis, which mainly determines the activity of mitochondrial respiration. PPARα, activated by PGC-1α, and AMPK enhance CPT1, the rate-limiting enzyme of FAO. The oncogene MYC also induces FAO through an as-yet-unknown mechanism. Through these mechanisms, increased OXPHOS and FAO produce sufficient ATP for cancer progression

The oncogene c-MYC (MYC) increases mitochondrial mass and FAO in specific cancer cell types. MYC activates mitochondrial biogenesis and fusion by upregulating PGC-1β and phospholipase D family member 6, respectively, to increase both the respiratory and biosynthetic capacity of cancer cells, supporting their rapid proliferation (Trotta and Chipuk 2017). MYC overexpression is associated with a poor prognosis in childhood neuroblastoma, while its inhibition resulted in cell death and neuronal differentiation in MYC-amplified neuroblastoma cells and increased the survival of MYC-transgenic mice (Zirath et al. 2013). These effects are attributed to the impairment of ETC and FAO, accompanied by intracellular lipid accumulation. MYC can deregulate FAO, and thus inhibition of FAO proved to have therapeutic efficacy in transgenic MYC-driven lymphoma (Pacilli et al. 2013). Likewise, FAO was shown to be characteristically elevated in MYC-overexpressing TNBC, and its inhibition suppressed tumorigenesis in a patient-derived xenograft model (Camarda et al. 2017). Despite the promise of these studies, the precise role of MYC in tumorigenesis requires validation in other cancer types, and therapeutic targeting of FAO should be further developed in known FAO-dependent MYC-overexpressing cancers (Fig. 2).

## Therapeutic exploitation of mitochondrial bioenergetics in oncology

As exemplified by the studies highlighted in the preceding sections, mitochondrial bioenergetics, which mainly consists of OXPHOS and FAO, is emerging as a novel and promising therapeutic target of cancer owing to its vital role in the cancer metabolism of malignant phenotypes. As part of extensive efforts devoted to the development of new anticancer drugs targeting cancer bioenergetics, numerous drugs related to these pathways are currently being tested in preclinical and clinical studies.

A drug delivery system that selectively targets mitochondria in cancer cells constitutes an important part of mitochondria-targeted therapeutics. Conjugation to lipophilic peptides or cations such as the triphenylphosphonium (TPP) cation is the most effective method developed to date (Kalyanaraman et al. 2018). For example, conjugation of TPP^+^ to metformin, which inhibits OXPHOS, enhanced the efficacy of the drug by about 1,000 times in treating an animal model of PDAC (Cheng et al. 2016). Several small-molecule compounds containing a TPP moiety have also displayed efficacy in both cancer cell lines and mouse models by suppressing OXPHOS, enhancing ROS production, and attenuating growth factor signaling. In addition, a mitochondria-targeted vitamin E analogue (mito-chromanol) exhibited selective cytotoxicity to breast cancer cells by depleting intracellular ATP.

Targeting mitochondrial biogenesis has demonstrated particular therapeutic efficacy for cancers of malignant phenotypes. For example, inhibition of mitochondrial biogenesis with gamitrinib, a mitochondrial HSP90 inhibitor, effectively eradicated BRAF-mutated melanoma cells, which were resistant to a MAPK inhibitor (Zhang et al. 2016). Gamitrinib also dramatically improved the efficacy of PI3K therapy for glioblastoma, overcoming its mitochondrial adaptation (Ghosh et al. 2015). Surprisingly, several FDA-approved antibiotics were also shown to effectively eradicate CSCs through the inhibition of mitochondrial biogenesis (Lamb et al. 2015). The endosymbiosis theory can explain these effects, which suggests that mitochondria originated from bacteria that had been engulfed by eukaryotic cells. Thus, ribosomes of mitochondria resemble those of prokaryotic cells, which can therefore be targeted by antibiotics.

OXPHOS inhibitors are emerging as promising therapeutics of malignant cancers marked by increased mitochondrial respiration (Wolf 2014). Antidiabetic biguanides, metformin and phenformin, have been rediscovered as OXPHOS inhibitors, and their anti-cancer effects have been proven in epidemiological, preclinical, and clinical studies (Lee et al. 2016c). Notably, metformin demonstrated selective efficacy against CSCs, sensitizing them to conventional treatments (Hirsch et al. 2009). The efficacy of biguanides largely depends on the reliance of cancer cells for OXPHOS, and the primary mode of action is suggested to involve interference of mitochondrial complex I (NADH dehydrogenase) (Birsoy et al. 2014). Recently, an integrated pharmacodynamic study determined the mode of action of metformin by investigating the metabolic adaptation responses in breast cancer patients (Lord et al. 2018). Two distinct groups were identified: an OXPHOS transcriptional response (OTR) group that upregulated the OXPHOS gene and an FDG response group that increased 18-FDG uptake. The OTR group displayed an increase in the proliferation signature and was resistant to metformin treatment. Nevertheless, only early results of clinical trials are currently available, limited to a few cancer types and surrogate outcome measurements (Chae et al. 2016). Clinical trials in non-diabetic primary breast cancer patients treated with neoadjuvant metformin exhibited clear improvement of cancer proliferation markers (Niraula et al. 2012). Clinical trials in pre-operative endometrial cancer patients with metformin monotherapy demonstrated a substantial decrease in the Ki67 proliferative index (Schuler et al. 2015). Since these data cannot provide a definite conclusion for clinical translation, survival outcomes of these trials are awaited. Regardless, metformin and other biguanides are clinically immediately exploitable drugs in selected cancer types owing to their proven safety with manageable toxicity profiles. Various other compounds (summarized in Table 1) have also been discovered to reduce cancer cell viability and metastasis by impairing OXPHOS and ATP production (Zhou et al. 2014; Lamb et al. 2014; Schöckel et al. 2015; Molina et al. 2018). For example, the FDA-approved antihelminthics nitazoxanide and niclosamide demonstrated efficacy against several cancer types, especially against CSCs, through inhibition of OXPHOS (Yo et al. 2012; Wang et al. 2013; Senkowski et al. 2015).

**Table 1:** Drugs with anticancer efficacy via targeting mitochondrial bioenergetics

Target	Drug	Cancer types	Developmental phase	References
Mitochondrial biogenesis				
Mitochondrial HSP90	Gamitrinib	Melanoma, glioblastoma	Preclinical	(Zhang et al. 2016; Ghosh et al. 2015)
Mitochondrial ribosome	Several antibiotics	Multiple types	Repositioning	(Lamb et al. 2015; De Luca et al. 2015)
Oxidative phosphorylation				
Mitochondrial complex I	Biguanides	Multiple types	Repositioning	(Birsoy et al. 2014; Hirsch et al. 2009)
	Rotenone	Glioblastoma	Repositioning	(Janiszewska et al. 2012)
	BAY 87-2243	Melanoma	Preclinical	(Schöckel et al. 2015)
	IACS-010759	AML and solid tumors	Phase 1 clinical	(Molina et al. 2018)
Mitochondrial complexes	Graphene	Multiple types	Preclinical	(Zhou et al. 2014)
MCT1/2	AR-C155858	Breast cancer	Preclinical	(Lamb et al. 2014)
Unknown	Nitazoxanide	Colorectal cancer	Repositioning	(Senkowski et al. 2015)
	Niclosamide	Breast cancer, Ovarian cancer	Repositioning	(Wang et al. 2013; Yo et al. 2012)
Fatty acid oxidation				
CPT1	Etomoxir	Multiple types	Repositioning	(Camarda et al. 2016; Lin et al. 2017; Tan et al. 2018)
	Perhexiline	Multiple types	Repositioning	(Rodriguez-Enriquez et al. 2015)
	ST1326	Lymphoma, leukemia	Preclinical	(Samudio and Konopleva 2015)
Unknown	Avocatin B	AML	Preclinical	(Lee et al. 2015b)
PPARα	NXT629	Melanoma, Ovarian cancer	Preclinical	(Stebbins et al. 2017)

The therapeutic efficacy of FAO inhibition has been proven in various types of cancer in preclinical studies. Etomoxir, a CPT1 inhibitor, is one of the most well-studied FAO inhibitors. Similar to metformin, etomoxir demonstrated selective efficacy against CSCs and cancers with malignant phenotypes, sensitizing them to conventional treatments (Camarda et al. 2016; Lin et al. 2017; Tan et al. 2018). Moreover, the combination of etomoxir with orlistat, which inhibits de novo FA synthesis, or mercaptoacetate, which inhibits lipolysis, resulted in synergistic effects (Li et al. 2013; Schlaepfer et al. 2014). Etomoxir has already been tested in clinical trials for patients with heart failure, but the trials have been retired due to the emergence of toxic side effects. Therefore, caution should be exerted when testing etomoxir in clinical trials on cancer patients by adjusting the dose or through consideration of the risk and benefit balance. Several other compounds (summarized in Table 1) have also demonstrated anticancer efficacy through inhibition of FAO, warranting further development (Lee et al. 2015b; Rodriguez-Enriquez et al. 2015; Samudio and Konopleva 2015; Stebbins et al. 2017).

Various combination strategies are now being attempted to make the best use of drugs targeting cancer bioenergetics. Combination of an OXPHOS/FAO inhibitor with other chemotherapeutic regimens has emerged as a particularly promising strategy. Mitochondrial respiration is a strong candidate to target the vulnerability of drug-resistant cancer cells, and several OXPHOS inhibitors can block their emergence. Various combinations of OXPHOS inhibitors with conventional cancer treatments—both cytotoxic and targeted therapies—proved to have synthetic lethality in pre-clinical models (Wolf 2014). For example, metformin potentiated a variety of chemotherapeutic agents, allowing for a decrease in the dose of chemotherapy and showing effectiveness against CSCs. Furthermore, the combination of a newly designed biguanide, HL156A (IM156), with temozolomide, a conventional therapeutic agent, demonstrated promising results in treating glioblastoma tumor spheres (Choi et al. 2016). Likewise, inhibition of FAO improved the efficacy of chemotherapeutic agents in various preclinical cancer models, including anti-androgen therapy against prostate cancer, cytarabine against leukemia, paclitaxel against breast cancer, sorafenib against CSCs of HCC, and rapamycin against several cancer types (Kuo and Ann 2018). Moreover, the association of mitochondrial targeting with molecular-targeted therapies is emerging as a novel strategy for determining effective drug combinations. Since numerous oncogenic kinase inhibitors create a dependence of surviving cells on mitochondrial metabolism, mitochondrial inhibition improved their efficacy in several preclinical studies (Marchetti et al. 2015). Inhibition of OXPHOS/FAO was also shown to increase the radiosensitivity of cancer cells in several preclinical models, which was mainly attributed to the alleviation of tumor hypoxia caused by decreased oxygen consumption (Gallez et al. 2017; Tan et al. 2018).

The concurrent inhibition of OXPHOS/FAO with other metabolic pathways is a fascinating approach to completely starve cancer cells to death. Typically, combined targeting of OXPHOS/FAO and glycolysis is an effective strategy. Inhibition of OXPHOS/FAO displayed efficacy against starvation-resistant cancer cells under glucose-deprivation conditions (Isono et al. 2016). The combination of metformin and 2DG was also proven to be effective against multiple cancer cell lines, tumor sphere models, and xenograft mouse models (Cheong et al. 2011; Kim et al. 2017). Inhibition of OXPHOS through suppression of PGC-1α led to loss of viability in melanoma cells, which was then rescued by HIF-1α-mediated activation of glycolysis (Lim et al. 2014). Dual suppression of PGC-1α and HIF-1α caused more severe energetic deficits and reduction of cell viability. Melanoma cells could still partially compensate for reduced cellular bioenergetics by glutamine utilization under dual inhibition of OXPHOS and glycolysis, while triple inhibition of PGC-1α, HIF-1α, and glutamine utilization completely blocked cell growth. Moreover, while glutaminolysis inhibition was insufficient to induce cell death in glutamine-dependent cancer, dual inhibition of FAO and glutaminolysis effectively induced cancer cell death (Halama et al. 2018). In addition, combined treatment of biguanide with gossypol, an aldehyde dehydrogenase (ALDH) inhibitor, demonstrated a remarkable therapeutic response in non-small cell lung cancer and glioblastoma tumor sphere models in vitro and in vivo (Park et al. 2018a). Since ALDH generates NADH that is fed to OXPHOS, this resulted in a significant reduction of ATP production.

## Concluding remarks: cancer bioenergetics-targeted strategy in cancer stem-like cells

Detailed studies on the metabolism and bioenergetics of cancer cells are shedding light for the next generation of cancer treatment in the postgenomic era. Although the advent of next-generation sequencing ushered in a huge wave of elucidation of the genome-level anomalies of cancer, resulting in numerous effective drug targets, the great diversity of these genes leaves us with an unsolved problem in this genomic era of effective targeting, especially for CSCs. Thus, the postgenomic era of cancer drug development should be driven by a paradigm shift toward bioenergetics, aiming at the energy metabolism distinctive to cancer cells. This strategy can directly target the fundamental vulnerability of cancer cells, with a focus on drug-resistant cancer cells.

Recently, adoption of a systems biology-based approach has effectively facilitated the discovery of feasible strategies to target cancer bioenergetics. For example, a recent study using an integrative analysis of signaling networks and drug functional networks revealed that subtypes of medulloblastoma with a poor prognosis can be targeted by digoxin (Huang et al. 2018). Specifically, digoxin treatment induced mitochondrial dysfunction in malignant subtypes of medulloblastoma, sensitizing the tumor cells to metabolic stress. Likewise, a chemical genomics approach to the development of drug repositioning for cancer therapy identified a novel candidate drug that specifically depletes crucial metabolite implicated in mitochondrial energy metabolism (Lee et al. 2016a). Thus, further applications of systems biology approach in the development of bioenergetics-targeted therapies is anticipated in the near future. For example, computational profiling of metabolic networks based on accurate quantification of metabolic fluxes enables identification of metabolic vulnerabilities in specific cancers (Hiller and Metallo 2013). Ultimately, for effective translation of these approaches, further validation is required extending to clinical trials of bioenergetics-targeted therapies.
